# Michelolide Enhances the Anticancer Efficacy of Radiation by Downregulating PD-L1 Protein Levels in Tumor Cells

**DOI:** 10.3390/ijms27114744

**Published:** 2026-05-25

**Authors:** Xuan Peng, Chunhua Tan, Yudie Shao, Dandan Li, Lu Li, Peizhong Kong

**Affiliations:** 1 Department of Biochemistry and Molecular Biology, School of Integrated Chinese and Western Medicine, Anhui University of Chinese Medicine, Hefei 230012, China; 17719371655@163.com (X.P.); 15290063428@163.com (Y.S.); 13083353061@163.com (D.L.); 2 Teaching and Research Section of Nuclear Medicine, School of Basic Medical Sciences, Anhui Medical University, Hefei 230031, China; tanchunhua224501@163.com; 3 Anhui Province Key Laboratory of Medical Physics and Technology, Center of Medical Physics and Technology, Hefei Institutes of Physical Sciences, Chinese Academy of Sciences, Hefei 230031, China; 4 Institute of Integrated Chinese and Western Medicine, Anhui Academy of Chinese Medicine, Hefei 230012, China

**Keywords:** breast cancer, radiotherapy, micheliolide, programmed death-ligand 1, antitumor immunity

## Abstract

Breast cancer is a type of cancer with the highest incidence and mortality rates among women. PD-L1 suppresses the proliferation and activation of T cells, thereby enabling cancer cells to evade immune surveillance and facilitating tumor progression. Micheliolide (MCL) is a guaianolide-type sesquiterpene lactone with broad biological activities. Our results revealed that radiation upregulates PD-L1 expression in breast cancer cells, while MCL pretreatment can inhibit this effect. Bioinformatics analysis combined with shRNA interference experiments confirmed that radiation upregulates PD-L1 by activating the IRF1-STAT1 signaling pathway, while MCL represses PD-L1 transcription by suppressing this pathway. In addition, MCL also downregulates PD-L1 protein level through accelerating proteasomal degradation of PD-L1. In vivo experiments demonstrated that MCL combined with radiotherapy significantly inhibits the growth of syngeneic tumors and increases intratumoral CD8^+^ T cell infiltration and the frequencies of granzyme B-positive cells. Taken together, our results indicate that MCL enhances T-cell-mediated antitumor immunity and improves radiotherapy efficacy through inhibiting IRF1-STAT1 signaling pathway-driven PD-L1 transcription and promoting PD-L1 protein degradation. This study provides a theoretical basis for the clinical application of MCL as an immunomodulator and radiosensitizer.

## 1. Introduction

Breast cancer is the most prevalent and lethal malignancy among women worldwide [1]. As a crucial therapeutic approach, radiotherapy (RT) is widely used in breast cancer treatment and significantly decreases the risk of local recurrence following breast-conserving surgery or mastectomy [2]. RT exerts anti-tumor effects mainly by directly inducing DNA damage-triggered tumor cell death, while anti-tumor immunity is also crucial for the tumor’s response to RT [3]. By reprogramming tumor immune microenvironment, RT can promote anti-tumor immunity and improve clinical outcomes. However, RT may also suppress anti-tumor immunity via multiple mechanisms, such as inducing the expression of immune checkpoint molecules and recruiting immunosuppressive cells [4,5]. Therefore, the combination of RT with anti-tumor immunomodulatory agents to synergistically enhance anti-tumor immunity represents a promising strategy for improving prognosis.

Programmed cell death ligand-1 (PD-L1), encoded by *CD274*, is a type I transmembrane glycoprotein which is located on the cell membrane or in the cytoplasm and sometimes in the nucleus [6,7]. Membrane-localized PD-L1 on cancer cells mediates tumor immune escape by binding to PD-1 on tumor-infiltrating lymphocytes [8]. Immunotherapies with anti-PD-1/PD-L1 antibodies have markedly improved therapeutic efficacy in various cancers [9]. Recently, increasing evidence shows that downregulating PD-L1 expression facilitates cancer treatment by boosting anti-tumor immunity [10,11,12]. The expression of PD-L1 is regulated at the multiple levels of transcription, post-transcription, and post-translation by multiple signaling pathways, including JAK/STAT, hypoxia-inducible factor-1α (HIF-1α), PI3K/Akt, and MAPK [13,14]. PD-L1 upregulation post-RT was demonstrated in several types of cancer, such as hepatocellular carcinoma, non-small-cell lung cancer (NSCLC), head and neck cancer, and colorectal cancer [15,16,17,18]. Mechanism research shows that signaling pathways including JAK/STAT, cGAS-STING, EGFR, and interferon gamma (IFN-γ) pathways are involved in ionizing radiation (IR)-induced PD-L1 expression [19]. Moreover, IR also upregulates PD-L1 expression by increasing its stability [20]. Considering the effect of IR on PD-L1 expression, it is reasonable to use agents that regulate PD-L1 expression as an adjuvant therapy for RT to enhance the therapeutic efficacy.

Micheliolide (MCL), a guaianolide-type sesquiterpene lactone, was first isolated from *Michelia compressa* and can be synthesized through the cyclization of parthenolide [21,22,23]. MCL exerts diverse biological activities, such as anti-inflammation, anti-tumor, antibacterial, anti-tissue fibrosis, and immunomodulatory function, by binding to nucleophilic biomolecules through its α-methylene-γ-lactone structure [22,24,25]. Studies indicated that MCL inhibits the proliferation of leukemia cells via irreversibly activating pyruvate kinase M2 (PKM2) by directly binding at residue cysteine424 (C424) [22]. In addition, MCL is well known as a nuclear factor kappa B (NF-κB) inhibitor and potently inhibits NF-κB-mediated inflammation and carcinogenesis [24,26]. Preliminary results of clinical trials (Registration Number: ACTRN12616000228482 and ChiCTR-OIC-17013604) revealed that ATC001/Dimethylaminomicheliolide (DMAMCL), the dimethylamino Michael adduct of MCL which releases MCL slowly and consistently, was well tolerated and exhibited anti-tumor activity in glioblastoma (GBM) patients. It is worth noting that ACT001 is able to inhibit PD-L1 transcription and trigger anti-tumor immune responses, which contributes to the therapeutic efficacy of ACT001 in GBM [25]. Our previous study demonstrated that MCL sensitized p53-deficient NSCLC to IR via promoting HIF-1α degradation in vitro [27]. However, the effect of MCL on PD-L1 expression after IR, as well as whether the combination of MCL and IR could enhance anti-tumor immunity and thereby improve treatment efficacy remain unclear.

In the present study, we demonstrated that MCL significantly inhibited IR-induced PD-L1 protein expression in multiple breast cancer cells. Furthermore, we found that MCL not only inhibited the activity of IRF1/STAT1 signaling axis-mediated PD-L1 transcription after IR, but also promoted the degradation of PD-L1 protein through the proteasome system. An in vivo study confirmed that the downregulating effect of MCL on PD-L1 protein level after IR is beneficial for increasing the infiltration of CD8^+^ T cells in tumor xenografts and enhancing the efficiency of RT. These results provide some hints that MCL may serve as an anti-tumor immune enhancer to improve the prognosis of breast cancer radiotherapy.

## 2. Results

### 2.1. Radiation Promotes PD-L1 Expression in Breast Cancer Cells

**Figure 1:**
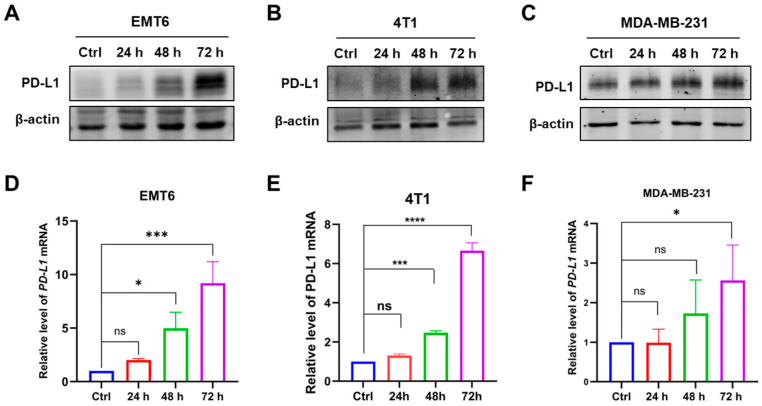
Expression of PD-L1 in breast cancer cells after irradiation. (**A**–**C**) PD-L1 protein levels of breast cancer cell lines (EMT6, 4T1, and MDA-MB-231) at different time points after radiation (4 or 8 Gy) treatment. (**D**–**F**) Relative mRNA levels of PD-L1 in breast cancer cells at different time points after irradiation (8 Gy). *: *p* < 0.05, ***: *p* < 0.001, ****: *p* < 0.0001, ns: not significant.

This study first examined changes in PD-L1 protein and transcriptional levels in breast cancer cells after exposure to a specific dose of radiation. Breast cancer cells (EMT6, 4T1, and MDA-MB-231) were treated with X-ray irradiation (4 or 8 Gy), and cellular proteins were collected at predetermined time points (24–72 h post-irradiation) for Western blot analysis. The changes in PD-L1 expression in breast cancer cells are shown in Figure 1A–C: in EMT6 cells, PD-L1 protein levels gradually increased from 24 h to 72 h after irradiation. By 72 h, PD-L1 expression was significantly upregulated compared to the control group (Ctrl group, non-irradiated cells). 4T1 cells exhibited a similar trend in PD-L1 upregulation as EMT6 cells. In MDA-MB-231 cells, only a slight increase in PD-L1 protein levels was observed between 24 h and 72 h post-irradiation. These results indicate that radiation can induce PD-L1 upregulation in multiple breast cancer cell lines, though the timing and extent of upregulation vary among different cell types.

Next, real-time quantitative PCR (RT-qPCR) was performed to assess PD-L1 transcriptional levels in irradiated breast cancer cells, aiming to elucidate how radiation upregulates PD-L1 protein expression in tumor cells. As shown in Figure 1D–F, PD-L1 mRNA levels gradually increased over time post-irradiation in breast cancer cells. By 72 h after irradiation, PD-L1 mRNA expression in all tested cell lines was significantly higher than that in their respective control groups (Ctrl group). These results demonstrate that radiation upregulates PD-L1 transcription in breast cancer cells.

### 2.2. MCL Suppresses Radiation-Induced PD-L1 Expression

**Figure 2:**
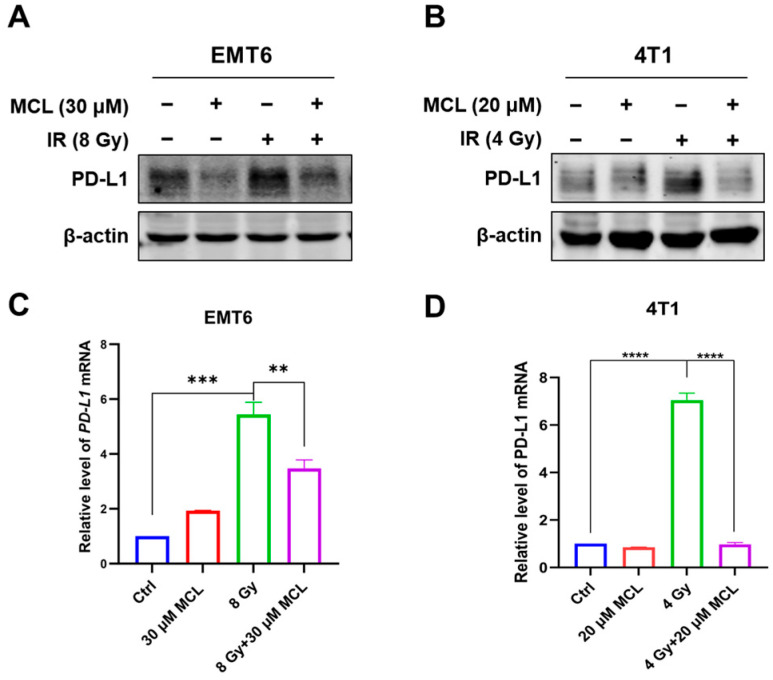
Effect of MCL on PD-L1 levels in breast cancer cells after radiation. (**A**,**B**) PD-L1 protein levels in EMT6 and 4T1 cells at 72 h after MCL and/or IR treatment. (**C**,**D**) Relative mRNA levels of PD-L1 in EMT6 and 4T1 cells at 72 h after MCL and/or IR treatment. **: *p* < 0.01, ***: *p* < 0.001, ****: *p* < 0.0001.

Next, this study examined the effect of MCL on PD-L1 expression after irradiation. Prior to X-ray irradiation (4 or 8 Gy) of breast cancer cells (EMT6 and 4T1), the cells were pretreated with MCL for 6 h. Total protein was then extracted at predetermined time points (72 h post-irradiation) for Western blot analysis. As shown in Figure 2A,B, MCL treatment alone at the indicated concentrations had minimal effect on PD-L1 protein levels in the tested cell lines. Irradiation significantly upregulated PD-L1 protein levels in all cell types, while MCL effectively suppressed this radiation-induced PD-L1 upregulation. In EMT6 and 4T1 cells, PD-L1 levels in the MCL+IR group were reduced to nearly baseline (comparable to their respective Ctrl groups). These results demonstrate that MCL potently inhibits radiation-induced PD-L1 protein expression in EMT6 and 4T1 cells.

The mRNA levels of PD-L1 in EMT and 4T1 cells were also measured by RT-qPCR after MCL and/or IR treatment. In EMT6 cells, MCL treatment alone moderately upregulated PD-L1 mRNA levels (the MCL group was approximately 1.9-fold that of the Ctrl group), but this effect was weaker than that induced by radiation (the IR group was approximately 5.4-fold that of the Ctrl group). Notably, MCL significantly reduced PD-L1 mRNA levels after irradiation (the MCL+IR group was approximately 3.4-fold that of the Ctrl group), with a statistically significant difference between the IR and MCL+IR groups (Figure 2C). The downregulatory effect of MCL on PD-L1 mRNA levels in irradiated 4T1 cells was consistent with that in irradiated EMT6 cells, while MCL treatment alone did not increase PD-L1 mRNA levels in 4T1 cells (Figure 2D).

Taken together, these results indicate that MCL effectively suppresses the radiation-induced upregulation of PD-L1 protein levels, and this inhibitory effect is partially attributed to MCL-mediated suppression of PD-L1 transcription following irradiation. Notably, MCL exhibited a stronger downregulatory effect on radiation-induced PD-L1 protein levels than on PD-L1 mRNA levels in EMT6 cells (Figure 2A,C). Such discrepancy may be attributed to multiple post-transcriptional regulatory mechanisms, including protein stability, translational regulation, and post-translational modification.

### 2.3. Bioinformatics Analysis Predicts Transcription Factors Regulating PD-L1

**Figure 3:**
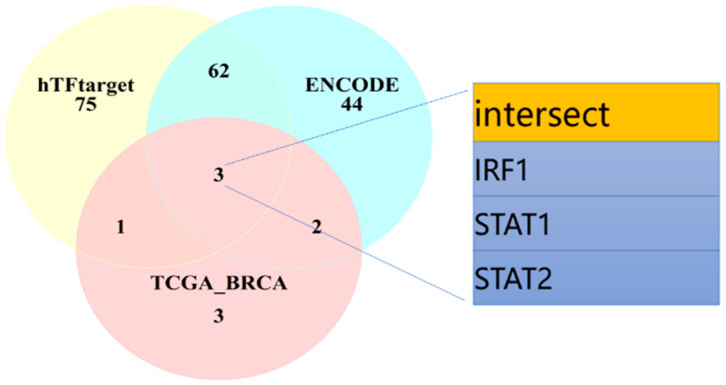
Bioinformatic screening of core transcription factors regulating PD-L1 expression in breast cancer. Three databases (hTFtarget, ENCODE, TCGA-BRCA) were used to mine PD-L1-regulating transcription factors, and IRF1, STAT1, and STAT2 were finally determined as key candidates via intersection analysis.

The previous research results have demonstrated that MCL regulates PD-L1 transcription after radiation. To further investigate the mechanism by which MCL modulates PD-L1, this study conducted bioinformatics analysis using three databases (hTFtarget, ENCODE, and TCGA) to screen for transcription factors (TFs) that regulate PD-L1 expression. In the hTFtarget and ENCODE databases, 141 and 111 TFs regulating PD-L1 expression were identified, respectively. Analysis of TCGA-BRCA data from the TCGA database revealed 9 PD-L1-associated TFs in breast cancer. Intersection analysis of these three datasets identified three key transcription factors closely related to PD-L1 expression in breast cancer: IRF1, STAT1, and STAT2 (Figure 3). The bioinformatics analysis results indicate that in breast cancer, both interferon regulatory factor 1 (IRF1) and STAT1 are important transcription factors regulating PD-L1 expression. Additionally, studies have shown that DNA double-strand breaks (DSBs), the most severe DNA damage induced by IR, can upregulate PD-L1 expression [15]. Following IR-triggered DSB formation, the phosphorylation levels of STAT1/3 and IRF1 are markedly elevated. The absence of IRF1 significantly attenuates IR-induced PD-L1 upregulation, suggesting that this process depends on the STAT1/3-IRF1 pathway [16]. Therefore, subsequent mechanistic studies will focus on IRF1 and STAT1.

### 2.4. The IRF1-STAT1 Signaling Axis Regulates Radiation-Induced PD-L1 Expression

**Figure 4:**
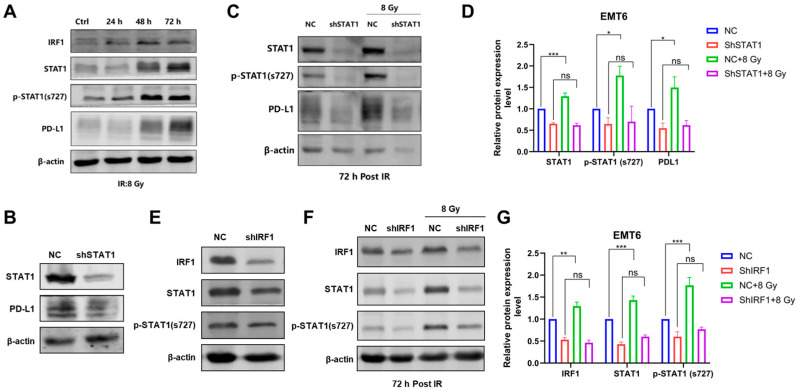
The mediating role of IRF1-STAT1 signaling pathway in radiation-induced PD-L1 expression. (**A**) Protein expression levels of p-STAT1/STAT1, IRF1 and PD-L1 in EMT6 cells at different time points after irradiation. (**B**) Expression levels of STAT1 and PD-L1 proteins in NC and shSTAT1 EMT6 cells. (**C**) Expression levels of p-STAT1/STAT1 and PD-L1 proteins in NC and shSTAT1 EMT6 cells after irradiation; Quantification of protein expression was depicted in (**D**). (**E**) Expression levels of IRF1 and p-STAT1/STAT1 proteins in NC and shIRF1 EMT6 cells. (**F**) IRF1 and p-STAT1/STAT1 protein expression levels in NC and shIRF1 EMT6 cells after irradiation; Quantification of protein expression was depicted in (**G**). β-actin was used as the loading control for normalization. *: *p* < 0.05, **: *p* < 0.01, ***: *p* < 0.001, ns: not significant.

Based on the aforementioned bioinformatics analysis results, this study further investigated the roles of IRF1 and STAT1 in radiation-induced PD-L1 expression. First, we examined the protein expression levels of PD-L1, STAT1, phosphorylated STAT1 (Ser727), and IRF1 in EMT6 cells at different time points after irradiation. The results showed that the expression of PD-L1, STAT1, phosphorylated STAT1 (Ser727), and IRF1 was upregulated post-irradiation, and the increasing trends in their expression levels were positively correlated (Figure 4A).

To determine the effect of p-STAT1(Ser727)/STAT1 on radiation-induced PD-L1 expression, we used shRNA to knock down STAT1 expression in EMT6 cells and established a stable STAT1-knockdown (shSTAT1) EMT6 cell line (Figure 4B). Negative control (NC) and shSTAT1 cells were irradiated, and the protein expression levels of p-STAT1(Ser727), STAT1, and PD-L1 were examined post-irradiation. As shown in Figure 4C,D, compared to the non-irradiated NC group, the levels of p-STAT1(Ser727), STAT1, and PD-L1 were significantly upregulated in irradiated NC cells. In contrast, in shSTAT1 cells, the expression levels of p-STAT1(Ser727), STAT1, and PD-L1 after radiation showed no significant difference compared to the non-irradiated group, indicating that p-STAT1(Ser727)/STAT1 mediates PD-L1 expression following irradiation.

To further determine whether IRF1 acts as an upstream regulator of p-STAT1(Ser727)/STAT1 expression after irradiation, we used shRNA to knock down IRF1 expression in EMT6 cells and established a stable IRF1-knockdown shIRF1 EMT6 cell line (Figure 4E). Negative control (NC) and shIRF1 cells were irradiated, and the protein expression levels of IRF1 and p-STAT1(Ser727)/STAT1 were examined following irradiation. As shown in Figure 4F,G, compared to the non-irradiated NC group, the levels of IRF1, p-STAT1(Ser727), and STAT1 were significantly upregulated in irradiated NC cells. In contrast, the radiation-induced expressions of IRF1, p-STAT1(Ser727), and STAT1 proteins were markedly attenuated in shIRF1 cells, indicating that IRF1 serves as an upstream regulator of p-STAT1(Ser727)/STAT1 expression following irradiation. In summary, the IRF1-STAT1 signaling pathway is involved in regulating radiation-induced PD-L1 expression.

### 2.5. MCL Downregulates PD-L1 Expression After Irradiation by Inhibiting the IRF1-STAT1 Signaling Pathway

**Figure 5:**
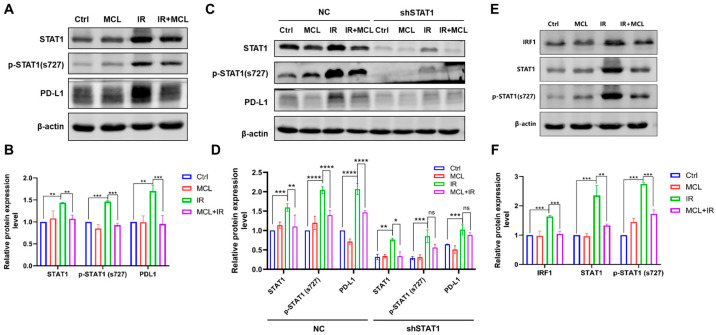
Regulatory effect of MCL on IRF1-STAT1 signaling pathway after irradiation. (**A**) Protein expression levels of p-STAT1/STAT1 and PD-L1 in EMT6 cells at 72 h after MCL and/or IR (8 Gy) treatment; Quantification of protein expression was depicted in (**B**). (**C**) Protein expression levels of p-STAT1/STAT1 and PD-L1 in NC and shSTAT1 EMT6 cells at 72 h after MCL and/or IR (8 Gy) treatment; Quantification of protein expression was depicted in (**D**). (**E**) IRF1 and p-STAT1/STAT1 protein expression levels in EMT6 cells at 72 h after MCL and/or IR (8 Gy) treatment; Quantification of protein expression was depicted in (**F**). β-actin was used as the loading control for normalization. *: *p* < 0.05, **: *p* < 0.01, ***: *p* < 0.001, ****: *p* < 0.0001, ns: not significant.

Furthermore, this study investigated whether MCL regulates radiation-induced PD-L1 expression through the IRF1-STAT1 signaling pathway. First, we examined the effect of MCL on p-STAT1(Ser727)/STAT1 levels after irradiation. The results showed that radiation significantly upregulated the protein expression of p-STAT1(Ser727) and STAT1 in EMT6 cells, while MCL partially suppressed such radiation-induced upregulation (Figure 5A,B).

Next, we assessed the impact of MCL on p-STAT1(Ser727)/STAT1 and PD-L1 protein expression after irradiation in STAT1-knockdown cells. As shown in Figure 5C,D, protein expression in NC cells was consistent with Figure 5A, whereas in shSTAT1 cells, radiation-induced upregulation of p-STAT1(Ser727), STAT1, and PD-L1 was effectively suppressed. Notably, in shSTAT1 cells, PD-L1 levels in the MCL+IR group remained comparable to those in the IR group, suggesting that p-STAT1(Ser727)/STAT1 is a key target of MCL regulation.

Additionally, we evaluated the effect of MCL on IRF1, the upstream regulator of STAT1. As demonstrated in Figure 5E,F, MCL downregulated IRF1 protein expression in irradiated cells compared with the IR group, along with suppressed downstream p-STAT1 and STAT1. In conclusion, MCL downregulates radiation-induced PD-L1 expression by inhibiting the IRF1-STAT1 signaling pathway.

### 2.6. MCL Promotes the Degradation of PD-L1 via the Proteasomal Pathway

**Figure 6:**
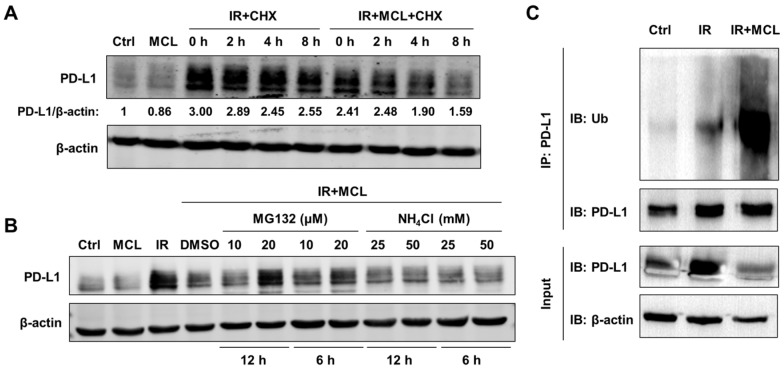
Effect of MCL on PD-L1 protein stability after irradiation. (**A**) CHX (100 μg/mL) was added to cells 72 h after MCL and/or IR (8 Gy) treatment, and the expression level of PD-L1 protein in EMT6 cells at different time points (2 h, 4 h, and 8 h) after CHX was added. The numbers marked between the bands represent the relative expression levels of PD-L1 protein in each group of cells. (**B**) Proteasome inhibitors (MG132, concentrations 10 μM and 20 μM) and lysosomal inhibitors (NH_4_Cl, concentrations 25 mM and 50 mM) were added to cells in the IR+MCL group at 72 h and 78 h after MCL and/or IR (8 Gy) treatment, respectively, to detect the expression levels of PD-L1 protein in each group at 84 h. (**C**) The ubiquitination level of PD-L1 protein in EMT6 cells was analyzed at 72 h after MCL (30 µM) and/or IR (8 Gy) treatment.

As previously mentioned, MCL regulates radiation-induced PD-L1 protein expression not only by modulating PD-L1 gene transcription but also through other post-transcriptional regulatory mechanisms that contribute to PD-L1 protein downregulation. Since changes in protein degradation rates significantly influence intracellular protein levels, this study investigated the effect of MCL on PD-L1 protein stability post-irradiation. The experiment utilized cycloheximide (CHX) to block intracellular PD-L1 synthesis, followed by monitoring PD-L1 levels over time to assess its degradation rate under different treatment conditions. As shown in Figure 6A, in the IR group, PD-L1 protein levels declined after CHX treatment, decreasing to 85% of the baseline (0 h) by 8 h. In contrast, in the IR+MCL group, PD-L1 levels dropped more rapidly, reaching only 66% of the baseline by 8 h, indicating that MCL accelerates the degradation of PD-L1 protein after irradiation.

To further explore the mechanism underlying MCL-mediated PD-L1 degradation, we investigated the two major intracellular protein degradation pathways: the proteasome and lysosome systems. MG132 (a proteasome inhibitor) and NH_4_Cl (a lysosome inhibitor) were used to block their respective pathways, and PD-L1 protein levels were subsequently analyzed. As demonstrated in Figure 6B, 20 μM MG132 effectively restored PD-L1 protein levels, with a more pronounced recovery observed at 12 h compared to 6 h, while NH_4_Cl had no impact on PD-L1 levels in the IR+MCL group. Moreover, the polyubiquitination level of PD-L1 obtained by immunoprecipitation (IP) assay was markedly elevated in the IR+MCL group relative to the IR group (Figure 6C). These results suggest that MCL promotes PD-L1 degradation primarily via the proteasome pathway.

### 2.7. MCL Enhances Anti-Tumor Immunity and Potentiates Radiotherapy-Induced Tumor Suppression

Based on the above in vitro experiment results, we further conducted in vivo studies for validation. Syngeneic tumor models were established by subcutaneously injecting EMT6 cells into the right hind limb of immunocompetent female BALB/c mice. When the tumor volume reached approximately 200 mm^3^, the mice were randomly divided into four groups: Ctrl group (DMSO), MCL group (intratumoral injection of MCL at 100 mg/kg/80 µL, administered every other day for a total of 5 injections), IR group (10 Gy local irradiation on day 0), and IR+MCL group (same treatment parameters as the MCL and IR groups). Day 0 was defined as the day of irradiation, and from day 0 onward, mouse body weight and tumor volume were measured every two days.

**Figure 7:**
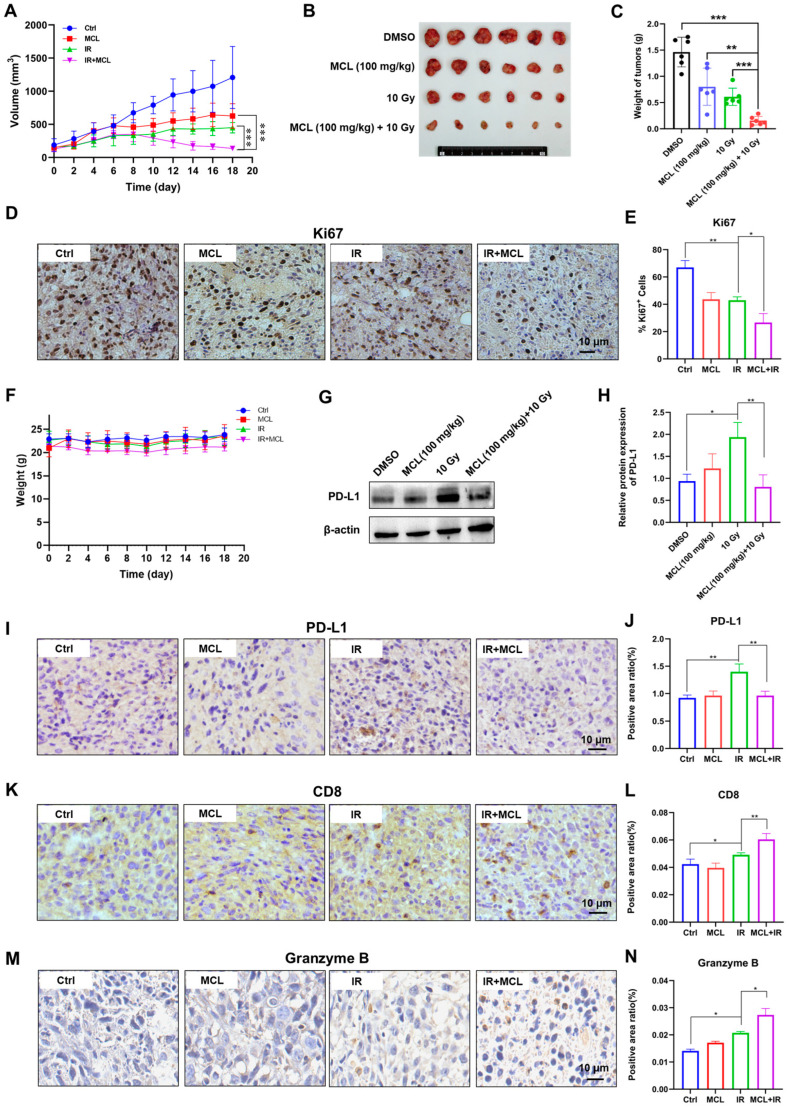
Effect of MCL on syngeneic tumor growth and intratumoral anti-tumor immune response following irradiation. (**A**) Changes in syngeneic tumor volume in each group (*n* = 6). (**B**) Syngeneic tumor volume monitoring termination time point (day 18 post-irradiation), pictures of stripped syngeneic tumors. (**C**) Monitoring the weight of the stripped syngeneic tumors at the termination time point (*n* = 6). (**D**) IHC assay demonstrated the expression of Ki67 protein in tumor tissues. Quantification of Ki67 positive cells was depicted in (**E**) (*n* = 3). (**F**) Changes in body weight of mice in each group (*n* = 6). (**G**) Western blot analysis of PD-L1 expression in tumor tissues from each group; Quantification of PD-L1 protein was depicted in (**H**) (*n* = 3). (**I**) IHC assay demonstrated the expression of PD-L1 protein in tumor tissues; Quantification of IHC staining of PD-L1 was in (**J**) (*n* = 3). (**K**) IHC assay demonstrated the expression of CD8 protein in tumor tissues. Quantification of IHC staining of CD8 was in (**L**) (*n* = 3). (**M**) IHC assay demonstrated the expression of Granzyme B protein in tumor tissues; Quantification of IHC staining of Granzyme B was in (**N**) (*n* = 3). *: *p* < 0.05, **: *p* < 0.01, ***: *p* < 0.001.

Tumor growth curves indicated that both MCL and radiation treatment alone suppressed tumor growth, while the combination of MCL and IR exerted the strongest inhibitory effect on tumor growth (Figure 7A). On day 18, mice were euthanized and tumors were excised, photographed, and weighed. Tumor images demonstrated that tumor volumes in the MCL and IR groups were smaller than those in Ctrl group, whereas the IR+MCL group exhibited the smallest tumor size (Figure 7B). Tumor weights were 1.46 ± 0.28 g (Ctrl), 0.80 ± 0.35 g (MCL), 0.61 ± 0.16 g (IR), and 0.16 ± 0.08 g (IR+MCL), with the IR+MCL group demonstrating significantly lower tumor weight than all other groups (Figure 7C). Immunohistochemistry (IHC) analysis of the proliferation marker Ki67 showed the highest proportion of Ki67-positive cells (darkly stained nuclei) in the Ctrl group, reduced levels in the MCL and IR groups, and the lowest proportion in the IR+MCL group (Figure 7D,E), consistent with the observed differences in tumor volume and weight. Throughout the experiment, body weight of mice fluctuations remained within normal ranges across all groups (Figure 7F).

Furthermore, we evaluated PD-L1 expression in tumor tissues by Western blot and IHC assay. Both detection methods confirmed that PD-L1 protein expression was upregulated in tumor tissues after irradiation, and MCL could significantly attenuate this upregulation, in line with in vitro experimental data (Figure 7G–J). We also investigated whether MCL affects T-cells-mediated antitumor immunity in syngeneic tumors. The IHC assay demonstrated that MCL remarkably enhanced CD8^+^ T cell infiltrations and increased the positive staining proportion of Granzyme B in irradiated tumors (Figure 7K–N). Together, these results indicate that MCL potentiates the antitumor efficacy of radiotherapy in vivo and enhances T-cell-mediated antitumor immunity.

## 3. Discussion

Cancer remains a major global health threat, accounting for approximately one-sixth of all deaths worldwide. It is fundamentally a complex disease caused by dysregulation of cell growth control [28,29,30]. Conventional treatments include surgery, radiotherapy, and chemotherapy, with radiotherapy being the most widely used, over half of cancer patients requires radiotherapy [31,32]. Modern radiotherapy techniques achieve precise tumor targeting, effectively killing cancer cells while maximizing protection of normal tissues and reducing treatment costs [33]. Current trends in radiotherapy development focus on two key directions: first, personalized treatment based on biological characteristics; second, combination with immunotherapy to enhance antitumor efficacy while reducing side effects, making it an important component of precision cancer medicine. These advances bring new hope for cancer treatment.

The PD-1/PD-L1 axis, as a critical immune checkpoint, has become an important target for cancer immunotherapy [34]. FDA-approved inhibitors such as nivolumab (anti-PD-1 antibody) and atezolizumab (anti-PD-L1 antibody) are now used to treat various cancers, including liver cancer [35,36]. However, primary or acquired resistance severely limits clinical efficacy, which is closely related to tumor heterogeneity and the complexity of the immune microenvironment [37]. Studies have shown that PD-L1 expression not only promotes immune escape but is also associated with enhanced cancer stemness [38,39]. Additionally, PD-L1 upregulation, combined with phenotypic changes in CD4^+^ T cells, leads to increased tumor aggressiveness and resistance to chemo/radiotherapy, ultimately affecting patient prognosis. These findings reveal the multifaceted role of PD-L1 in tumor drug resistance.

Micheliolide (MCL) is a naturally occurring sesquiterpene lactone compound capable of crossing the blood–brain barrier, demonstrating broad potential in anti-inflammatory and antitumor applications [40,41,42,43]. It significantly inhibits PD-1/PD-L1 expression, restores T-cell function, and blocks tumor immune evasion [44]. Its antitumor mechanisms primarily involve regulation of multiple radiation resistance-related pathways: by binding IKKβ to inhibit NF-κB activation and promote ROS generation; targeting AEBP1 to block the PI3K/Akt pathway and suppress cancer stem cell activity; and directly binding STAT3 to inhibit its phosphorylation, downregulate PD-L1 expression, and enhance T-cell immune responses [45,46,47]. These properties make it particularly effective against refractory tumors, offering a novel therapeutic strategy to overcome radioresistance [25].

This study found that radiation treatment alone significantly upregulated PD-L1 protein expression in breast cancer cells (MDA-MB-231, EMT6, and 4T1). Further analysis revealed that PD-L1 transcription levels also increased post-radiation in some tumor cells, suggesting radiation may promote PD-L1 expression through transcriptional regulation. Notably, micheliolide (5 µM-30 µM) effectively suppressed radiation-induced (8 Gy) PD-L1 protein expression in EMT6 and 4T1 cells, laying a molecular foundation for improved anti-tumor effects of radiotherapy. This finding aligns with previous research, further supporting MCL’s potential in improving radiotherapy outcomes [27].

Mechanistic studies using gene knockdown confirmed that radiation upregulates PD-L1 expression in tumor cells via activation of the IRF1/STAT1 signaling pathway. Further investigation demonstrated that MCL significantly reduces radiation-induced PD-L1 expression by inhibiting the IRF1/STAT1 pathway. Preliminary studies also suggest MCL may promote PD-L1 protein degradation via the ubiquitin-proteasome pathway. In addition to ubiquitination, common post-translational modifications such as glycosylation, phosphorylation and lactylation are critical for regulating PD-L1 protein stability. Glycosylation can protect PD-L1 from proteasomal degradation and maintain its membrane expression [12]. Site-specific phosphorylation prevents PD-L1 degradation, drives PD-L1 aggregation and impairs cancer immune responses [48]. Lysine lactylation of PD-L1 at the K280 residue within its intracellular domain stabilizes PD-L1 via blocking the binding of E3 ubiquitin ligase HUWE1, thereby suppressing its ubiquitination and subsequent proteasomal degradation [49]. Considering the regulatory complexity of PD-L1, exploring how MCL mediates the above modifications under radiation will be one of our key research directions in the follow-up work.

In syngeneic tumor models, immunohistochemistry confirmed that MCL markedly suppresses PD-L1 protein expression, enhances CD8^+^ T cell infiltrations and increases the frequencies of granzyme B-positive cells in irradiated tumors. Additionally, MCL effectively inhibited syngeneic tumor growth and demonstrated potential in activating T cells-mediated antitumor immune responses.

## 4. Materials and Methods

### 4.1. Cell Culture and Reagents

The breast cancer cell lines EMT6, 4T1, and MDA-MB-231 used in this study were purchased from the Cell Bank of Type Culture Collection of Chinese Academy of Sciences (Shanghai, China). EMT6 and 4T1 cells were cultured in RPMI-1640 medium (KeyGEN BioTECH, Nanjing, China), while MDA-MB-231 cells were cultured in DMEM medium (KeyGEN BioTECH). All media were supplemented with 10% FBS (Lonsera, Montevideo, Uruguay), 100 μg/mL streptomycin (Gibco, Carlsbad, CA, USA), and 100 U/mL penicillin (Gibco). Cells were maintained at 37 °C in a humidified incubator with 5% CO_2_. All cells underwent STR profiling and were confirmed to be free of mycoplasma and other contaminants. MCL was purchased from Wuhan ChemFaces Biochemical Co., Ltd. (Wuhan, China). Cycloheximide (HY-12320), MG132 (HY-13259), and Ammonium chloride (NH_4_Cl, HY-Y1269C) were sourced from MedChemExpress (MCE, Shanghai, China).

### 4.2. Animals

Female BALB/c mice aged 6–8 weeks were purchased from Hangzhou Ziyuan Animal Technology Co., Ltd. (Hangzhou, China). Mice were housed in a specific pathogen-free (SPF) environment with free access to water and feed. All animal experiments were approved by the Ethical Committee of Experimental Animals of Hefei Institutes of Physical Science, Chinese Academy of Sciences (Code of Ethics: SWYX-DW-2021-13).

### 4.3. Cell Irradiation Treatment

Cells were irradiated with an XHA600D X-ray irradiator (SHINVA, Zibo, China) at a dose rate of 0.189 Gy/min. In the case of combined treatment with MCL and IR, cells were pretreated with MCL for 6 h before irradiation.

### 4.4. Western Blot and Immunoprecipitation

Cells were lysed in RIPA buffer (P0013B, Beyotime Biotechnology, Shanghai, China) containing 1 mM PMSF (ST505, Beyotime Biotechnology). Protein samples were separated by SDS-PAGE and transferred to PVDF membranes. After blocking with 5% skim milk for 1 h at room temperature, membranes were incubated with primary antibodies overnight at 4 °C. Primary antibodies used in this study were as follows: anti-PD-L1 Mouse (ab213480; Abcam, Cambridge, UK), anti-PD-L1 Human (ab213524; Abcam), anti-IRF1 (11335-1-AP; Protein Tech Group, Wuhan, China), anti-STAT1 (240132; ZenBio, Chengdu, China), anti-p-STAT1(Ser727) (R25797; ZenBio), anti-ubiquitin (10201-2-AP; Protein Tech Group), and anti-β-actin (HA601082; HuaBio, Hangzhou, China). After extensive washing with TBST (Tris-HCl buffer with 0.1% Tween-20), membranes were incubated with IRDye800^®^-conjugated anti-rabbit (926-32211, LI-COR Biosciences, Lincoln, NE, USA) and IRDye^®^680 DX-conjugated anti-mouse (926-68070, LI-COR Biosciences) secondary antibodies for 1 h at room temperature. Immunoreactive bands were imaged using an Odyssey^®^ CLx Imaging System (9140-00, LI-COR Biosciences). Protein quantification was performed using ImageJ 1.x software (National Institutes of Health, Bethesda, MD, USA).

For immunoprecipitation (IP), 2 μg of anti-PD-L1 antibody (Abcam) incubated with cell lysate, followed by incubating with protein-A/G agarose (Beyotime Biotechnology). The beads were washed extensively and then suspended in SDS loading buffer for Western blot analysis.

### 4.5. RNA Extraction and Quantitative Real-Time PCR

Total RNA was isolated using AG RNAex Pro Reagent (A6A4730, Accurate Biotechnology, Changsha, China), and cDNA was synthesized from 2 μg RNA using a reverse transcription kit (11123ES60, Yeasen, Shanghai, China). Quantitative PCR (qPCR) was performed on a Roche 480 Light Cycler (05015278001, Roche, Basel, Switzerland) with Hieff qPCR SYBR Green Master Mix (11201ES08, Yeasen). The primers used for PCR amplification are shown as follows: 5′-TGCGGACTACAAGCGAATCACG-3′, 5′-CTCA GCTTCTGGATAACCCTCG-3′ (Mouse *PD-L1*), 5′-GGACAAGCAGTGACCATCAA-3′, 5′-GTGTGCTGGTCACATTGAAAA-3′ (Human *PD-L1*), 5′-CTGGGACGACATGGA GAAAA-3′, 5′-AAGGAAGGCTGGAAGAGTGC-3′ (Mouse *ACTB*) and 5′-CATGTACG TTGCTATCCAGGC-3′, 5′-CTCCTTAATGTCACGCACGAT-3′ (Human *ACTB*). *ACTB* was used as a normalizing control. Data were analyzed using the 2^−ΔΔCt^ method.

### 4.6. Lentiviral Infection

The RNA interference silencing plasmids pLV3-U6-STAT1-shRNA-Puro and pLV3-U6-IRF1-shRNA-Puro were generated by subcloning mouse STAT1- and IRF1-targeting short hairpin RNA (shRNA) oligonucleotide sequences into the lentiviral vector pLV3-U6 (Miaoling Biotechnology, Inc., Wuhan, China). The shRNA target sequences of STAT1 and IRF1 were CTGTGATGTTAGATAAACA and GCTAGAGATGCAGATTAATTCT, respectively. EMT6 cells were infected with the prepared lentivirus, and puromycin (2 mg/mL) was added 48 h post-infection for 10 consecutive days to generate the stable STAT1 and IRF1 knockdown cell lines.

### 4.7. Syngeneic Model and Treatments

Syngeneic tumor models were established by subcutaneously injecting 5 × 10^5^ EMT6 cells into the right hind limb of female BALB/c mice. When the tumor volume reached between 100 and 200 mm^3^, the mice were randomly assigned to four groups (each group contained six mice): Control group (Ctrl): treated with DMSO; MCL group: received intratumoral injection of MCL at 100 mg/kg in 80 µL solution, once every other day for a total of 5 injections; IR group: subjected to 10 Gy local irradiation of the tumors on day 0; IR+MCL group: administered with MCL and irradiation using the same parameters as the MCL and IR groups, respectively. The day of irradiation was defined as day 0. Body weight and syngeneic tumor volume were measured every two days. The calculation of tumor volume was as follows: (L × S^2^)/2. L represents the length of the longest diameter, and S represents the length of the shortest diameter. The mice were sacrificed 18 days after irradiation, and all tumors were excised.

### 4.8. Immunohistochemistry

The excised syngeneic tumors were fixed in 10% formalin and embedded in paraffin. The embedded tumor tissues were cut into 5 μm sections, which were subjected to dewaxing and rehydration, followed by antigen retrieval and blocking. These prepared slides were incubated overnight with primary antibodies as follows: anti-Ki67 (28074-1-AP; Protein Tech Group), anti-PD-L1 (ab213480; Abcam), anti-CD8A (A23305PM; ABclonal, Wuhan, China) and anti-Granzyme B (13588-1-AP, Protein Tech Group). After washing, slices were incubated with HRP-conjugated Goat Anti-Rabbit IgG(H+L) (SA00001-2; Protein Tech Group) for 30 min. Then, DAB chromogenic detection and hematoxylin counterstaining were performed, and the expression of the target proteins was observed and photographed under a microscope.

### 4.9. Statistical Analysis

All statistical analyses were performed using GraphPad Prism software (v9.31). Quantitative data were presented as mean ± standard deviation (SD). Experiments were repeated at least three times independently. Two-group comparisons were performed using unpaired Student’s *t*-test. One-way analysis of variance (ANOVA) followed by Duncan’s multiple range tests was applied for comparisons among multiple groups. Significance levels: * *p* < 0.05, ** *p* < 0.01, *** *p* < 0.001, **** *p* < 0.0001; ns: not significant.

## 5. Conclusions

In summary, the findings of this study demonstrate that micheliolide (MCL) significantly downregulates PD-L1 protein levels in breast cancer cells, exhibiting potential to activate anti-tumor immunity. By investigating MCL’s regulatory effects on radiation resistance-related signaling pathways (e.g., IRF1 and STAT1 pathways) and key proteins (e.g., PD-L1), we elucidated its underlying mechanisms. This study provides a theoretical basis for the clinical application of MCL as an immunomodulator and radiosensitizer.

## Data Availability

The original contributions presented in this study are included in the article. Further inquiries can be directed to the corresponding authors.

## Abbreviations

The following abbreviations are used in this manuscript:

PD-L1	Programmed cell death ligand 1
MCL	Micheliolide
IR	ionizing radiation
DMSO	Dimethyl Sulfoxide
STAT1	signal transducer and activator of transcription 1
IRF1	Interferon Regulatory Factor 1
DSB	DNA double strand break
PD-1	programmed cell death protein 1
PTL	Parthenolide
